# Identifying Sleep-Deprived Authors of Tweets: Prospective Study

**DOI:** 10.2196/13076

**Published:** 2019-12-06

**Authors:** Sara Melvin, Amanda Jamal, Kaitlyn Hill, Wei Wang, Sean D Young

**Affiliations:** 1 University of California Institute for Prediction Technology Los Angeles, CA United States; 2 Department of Medicine University of California, Los Angeles Los Angeles, CA United States; 3 New York University-Winthrop Hospital Mineola, NY United States; 4 Department of Computer Science University of California, Los Angeles Los Angeles, CA United States; 5 Department of Medicine University of California, Irvine Orange, CA United States; 6 University of California Institute for Prediction Technology Irvine, CA United States

**Keywords:** wearable electronic devices, safety, natural language processing, information storage and retrieval, sleep deprivation, neural networks (computer), sleep, social media

## Abstract

**Background:**

Social media data can be explored as a tool to detect sleep deprivation. First-year undergraduate students in their first quarter were invited to wear sleep-tracking devices (Basis; Intel), allow us to follow them on Twitter, and complete weekly surveys regarding their sleep.

**Objective:**

This study aimed to determine whether social media data can be used to monitor sleep deprivation.

**Methods:**

The sleep data obtained from the device were utilized to create a tiredness model that aided in labeling the tweets as sleep deprived or not at the time of posting. Labeled data were used to train and test a gated recurrent unit (GRU) neural network as to whether or not study participants were sleep deprived at the time of posting.

**Results:**

Results from the GRU neural network suggest that it is possible to classify the sleep-deprivation status of a tweet’s author with an average area under the curve of 0.68.

**Conclusions:**

It is feasible to use social media to identify students’ sleep deprivation. The results add to the body of research suggesting that social media data should be further explored as a potential source for monitoring health.

## Introduction

### Background

Sleeping fewer than 7 hours per night (ie, sleep deprivation) has been associated with a large number of public health concerns, including elevated blood pressure, weight gain, impaired glucose tolerance, type 2 diabetes mellitus, increased anxiety levels, and cardiovascular disease [1-6]. Poor sleep has also been associated with cognitive and motor performance deficiencies, which can lead to car accidents [7], plane crashes [8,9], and medical errors [10]. Unfortunately, the prevalence of sleep deprivation has increased by 31% (31/100) from 1985 to 2012 [11]. In 2014, 34.8% (348/1000) of US adults received, on average, 6 hours of sleep or less in a 24-hour period [12].

Sleep deprivation is difficult to measure because of limited measurement tools. Owing to the difficulty in recruiting participants to sleep in a sleep laboratory for long periods where they can be objectively studied, most sleep studies typically use self-reported items that carry subjective bias [13,14]. For example, according to the Behavioral Risk Factor Surveillance System, during 2009-2010, an estimated 1 in 25 adult drivers (aged 18 years or older) reported having fallen asleep while driving in the previous 30 days, suggesting limitations in self-reports of tiredness as people are not aware of their tiredness state or the impact it may have on their performance [15,16]. Although more objective technological advancements such as actigraphy and home polysomnography are available for use in research, there are limitations in applying these research-grade devices in large epidemiological and real-world settings. Therefore, new methods and tools are needed to help determine whether and when a person is sleep deprived.

Consumer-wearable smart watches and social media might be able to be used as an easy-to-integrate and more objective measure to monitor whether people are sleep deprived [17]. Unlike research-grade sleep-tracking devices, consumer wearables have the potential to monitor sleep in an unobtrusive way as consumers are naturally wearing them in daily life. However, a limitation of wearable devices is that people often choose to not wear them, thereby reducing the ability to gain sleep-tracking data.

In addition, one could use mobile phone to track their sleep; however, for many mobile phone sleep apps to work successfully, the phone has to stay on the bed the whole night to measure, there can only be one person in the bed, and the phone has to have sufficient amount of power to run an app throughout the night. Therefore, social media, which has been found to be useful as a tool for remote monitoring of behaviors, might be able to address this limitation and be used as an additional sleep monitoring tool. For example, researchers have already shown it is possible to mine text data within social media sites such as Facebook and Twitter to monitor and predict health outcomes, such as foodborne illness, influenza outbreaks, and HIV [18-20], and to monitor health behaviors [21-25]. Social media text might be similarly mined and studied to monitor sleep deprivation.

In addition, studies support that language skills appear to be affected by sleep deprivation, providing further support to our hypothesis that social media text data could be used to identify sleep deprivation [26]. For example, Harrison and Horne showed that sleep-deprived individuals generate fewer words and use less novel word associations when performing a word association task [27]. Therefore, it was hypothesized that linguistic features could passively and nondirectly characterize the tiredness state of the author at the time they created the text.

### Objective

This study sought to determine the feasibility of integrating wearable smart watches and social media to monitor and verify sleep deprivation among first-year students in college. We further explored whether wearable device data and social media data could be used as tools for remotely monitoring *tiredness*. We hypothesized that students would tweet differently when they were sleep deprived compared with when they were not and that tweet data could therefore be used as a method for identifying sleep deprivation among students. Our method leverages the use of machine learning in sleep-deprivation linguistic characteristics in digital communications [27]. Machine learning algorithms have been used in several areas of health [20]; however, these methods have not been previously applied to sleep research.

## Methods

### Study and Participants

In 2015, between October and December, 197 first-year undergraduate students from the University of California Los Angeles (UCLA) enrolled to participate in a study aimed to analyze sleep and stress patterns among university undergraduates. Students were targeted for this study because they were a convenience sample and would provide the necessary data; 94.1% (941/1000) of college students use social networking sites [28], and the average college student spends 94.6 min per day doing various mobile phone activities, such as checking their social media networks, texting, and checking or sending emails [29].

To qualify for the study, students had to meet the following criteria: be 18 years of age or older and younger than 21 years of age, be a first-year or first-year transfer student, be in their first semester at UCLA, and have at least three posts per week on Twitter. Students self-reported these criteria, and then a research assistant verified their student status through their student ID card and verified their Twitter use by accessing their Twitter profile. The participants allowed us to follow them on Twitter to collect their tweets during the course of this study. Subjects were provided US $5 for each completed survey and an additional US $5 if all surveys were completed in a month. The total was disbursed to students after the study ended in the form of an Amazon gift card. The UCLA institutional review board approved the study protocol.

Students were asked to wear an Intel Basis sleep monitoring device, allow us to follow them on Twitter, and complete weekly Web-based surveys to self-assess psychological and sleep health, including sleep quality, stressors that week, ability to deal with these stressors, and their emotions. Owing to the subjective nature of sleep deprivation [30], several scales have been used in the clinical and scientific community to clarify the definition of sleep deprivation. These surveys assessed perceived sleep deprivation by determining the quality of sleep during the previous week and the previous night on a 5-point Likert scale of very bad, bad, average, good, and very good. Out of the 197 students who originally signed up for the study, 86 students tweeted at least once, took at least one survey, and got at least one read on their smart wristband. However, only 64 students consistently tweeted every week throughout the study. All 86 student tweets had 17,889 unique words where the average word (which includes URLs, hashtags, and mentions) length was 10.023 (SD 6.416).

Finally, participants’ tweets were gathered while they were enrolled in the study using Twitter representational state transfer application program interface.

### Data Classification

The goal of this study was to use Twitter data to create a model to classify whether a tweet was made by a person who was sleep deprived at the time it was posted. To accomplish this goal, we first had to develop a data processing method to properly label every tweet as to whether the author was sleep derived at the time of the post or not. Then, a model had to be trained to classify these tweets to their correct category.

#### Data Preprocessing

The concept of *tiredness* is a complex notion that rises and falls throughout the day depending on a variety of factors such as quantity of sleep the night before. The Intel Basis bands provided minute-level sleep-tracking data, allowing us to use it to estimate an initial model of how tired a person is throughout their day. On the basis of the work by Pressman [31], we defined sleep deprivation as sleeping for fewer than 6 hours within 24 hours.

A sleep-labeling algorithm was created based on a simple linear model (f(x)=mx+b), where the start of every line begins at the end of a *new-day sleep*. A *new-day sleep* refers to the sleep duration that starts on one day and ends on the next day, or it starts the day after the last new-day sleep ended. All nap durations that were within that new-day plus the new-day sleep total duration were combined for the total estimate of sleep duration after the new-day sleep. Therefore, a student with a total amount of sleep greater than 6 hours (360 min) at the end of a new-day sleep was seen as starting out their day with a tiredness level (TL) of zero. This resetting is based on Pressman’s [31] work on sleep deprivation and is a simplification of the real-world tiredness model; however, our method will show to be sufficient for our needs. Any other duration of sleep less than 6 hours started out with a TL of 360-γ, where γ represents the total amount of the student’s sleep in minutes. Therefore, the equation to determine the minimum TL after a new-day sleep is max (360-γ,0).

A simple linear model is used to describe a person’s cognitive ability as a person grows more sleep deprived. This type of model was used because Dawson and Reid [32] showed a form of linear digression of a person’s cognitive performance over a period, where at 16 hours, it is equivalent to the performance of a person with a 0.02 g/dL blood alcohol level, which, for context, is greater than the US California blood alcohol limit for a person younger than 21 years. Finally, the TL threshold for sleep deprivation is considered 360 because if a person gets the minimum amount of sleep (ie, 1 min of sleep), then they will start out with the maximum TL and will be considered sleep deprived (SLD). Therefore, the slope of every linear segment is then m=(max_TL_-min_TL_)/(t_SLD_-t_wake_) where max_TL_ is the maximum TL after a subject has been awake for over 16 hours after a perfect new-day sleep, min_TL_ is the minimum TL after a perfect new-day sleep, t_SLD_ is the amount of time it takes, in minutes, for a person to become sleep deprived after a perfect amount of sleep, and t_wake_ is the amount of time, in minutes, that has lapsed since the start of the new-day sleep. The min_TL_ after a perfect night’s sleep will always be zero. In addition, the time lapsed after a person just woke up will always be zero as well. Therefore, every linear segment will have the slope of m=(max_TL_-min_TL_)/(t_SLD_-twake)=360/(16*60)=3/8.

**Figure 1:**
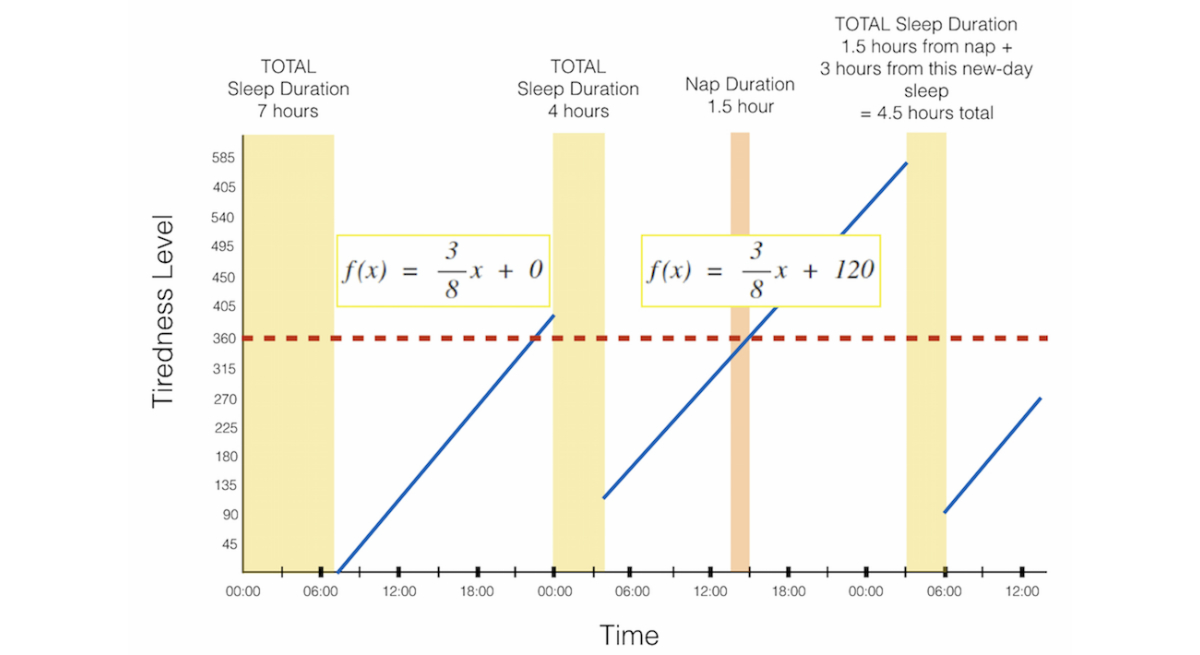
This shows a numeric model example of a student’s tiredness throughout their day. To label student tweets at any given minute, a simple tiredness model was created to help define when an author of a post was sleep deprived and when they were sleep sufficient. If a tweet was posted during the time a person was in their sleep deprived state (during the moment in time that the linear segment was above a tiredness level of 360), those tweets were labeled as sleep deprived while all others were labeled as sleep sufficient.

For example, as portrayed in Figure 1, a student sleeps a full 7 hours. As this sleep is the first sleep, we assume that it is the *new-day* sleep and start the tiredness model’s linear segment when the student wakes up that morning at 07:00. The student received 7 hours of sleep (420 min) the first night; thus, the y-intercept variable in f(x)=(3/8) x+b is max(360-420,0)=0. After 16 hours, the student is considered sleep deprived until they fall asleep for another new-day sleep, which approximately happens between the hours of 23:00 and 00:00 in this example.

Any tweets during the student’s sleep-deprived time will be labeled as *sleep deprived*, whereas all tweets that occurred between the hours of 07:00 and 23:00 will be labeled as *sleep sufficient*.

The start of the student’s next sleeping period occurs on the next day after the student wakes up from their last new-day sleep. Therefore, the second sleeping period shown in this example is considered a new-day sleep. The student, in this example, received only 4 hours of sleep (240 min), thus the y-intercept variable in f(x)=(3/8) x+b is max(360-240,0)=120. The student did not sleep enough that night, so they took a nap around 13:30 for approximately 1.5 hours. As this sleep does not start a day after the last new-day sleep occurred nor does this sleeping period end in the next day, this period of rest is considered a nap and will be added to the following new-day sleep.

Therefore, the time this student was sleep deprived, based upon our definition, would be between 14:40 and 03:00 the next day, when the student started their next new-day sleep. Any tweets by that student between that period will be labeled as *sleep deprived*.

#### Data Classification Model

After the data are labeled, we can then use supervised models to classify whether a student is sleep deprived or not based upon their Twitter posts. Out of all supervised methods, gated recurrent unit (GRU) [33], a type of recurrent neural network, was chosen to classify tweets based upon its abilities to take a tensor as an input and to consider the ordering of words into the calculation of the final classifier [34]. These key unique characteristics of the GRU are what aided this model’s ability to prove its superior performance compared with other supervised methods.

**Figure 2:**
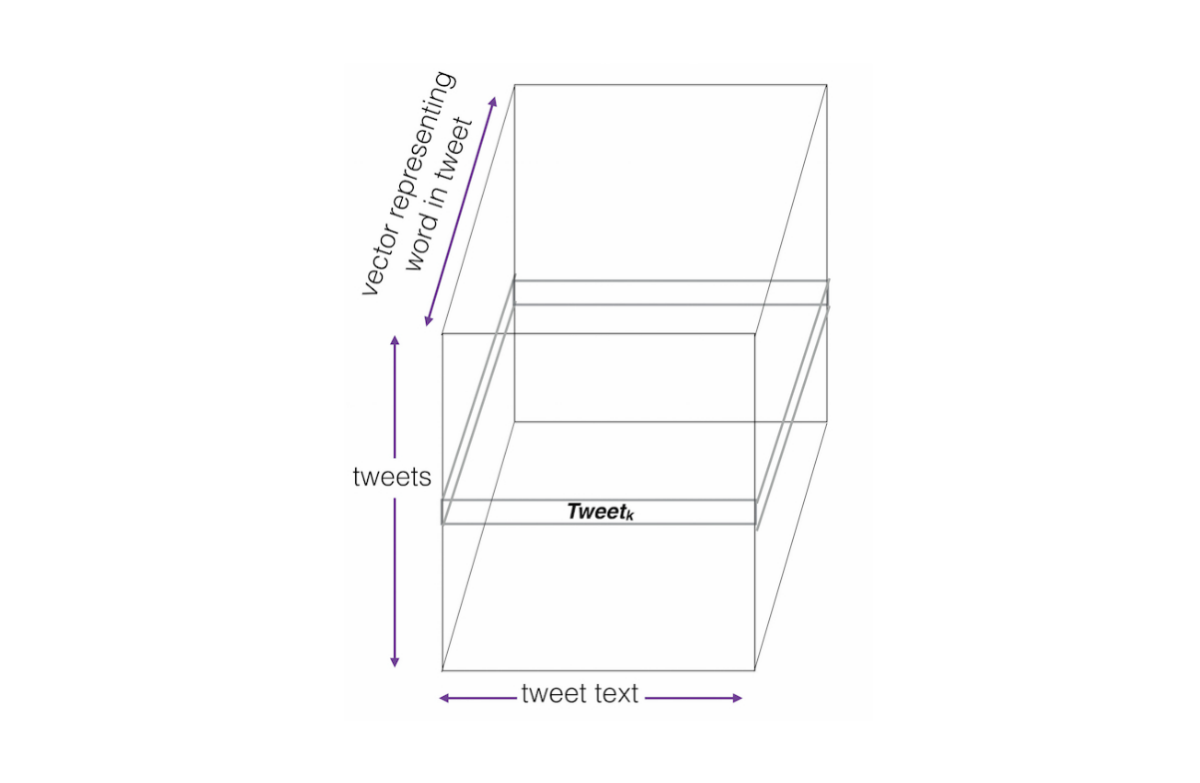
Input tensor description of each dimension. Each row of the input tensor represents a tweet while each column of the tensor is a word in the tweet (not including stopwords or non-English characters). Lastly, the third dimension is the numeric vector representation of a word concatenated with the sentiment of the tweet, the subjectivity of the tweet, and the hour the tweet was posted.

For most supervised methods, the input must be a matrix in the domain 
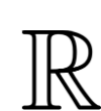
*^nxm^*. Originally, this study created an input matrix in the domain 
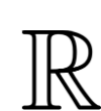
*^nxm^* where each row represents a tweet and each element (ie, word) in the row is represented by the bag-of-words method. The bag-of-words model counts frequency of terms and does not consider the order of the words or their similarity in meaning to other words. Therefore, to add correspondence of word meaning while also maintaining word ordering, the input matrix was extended to an input tensor. Similar to the input matrix in the domain 
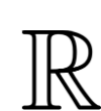
*^nxm^*, each row represents a tweet. However, instead of a bag-of-words term frequency representation of a word, each word is represented by a word vector, and the ordering of the words in the sentence is maintained. Hence, the input was represented by a tensor I in the domain 
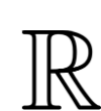
^δxκxξ^ where δ is the number of tweets, κ is the maximum tweet length of all the posts in the dataset, and ξ is the length of the word vectors used plus 3. The additional 3 is to account for the concatenation of the hour of the post, the sentiment of the post, and subjectivity of post integers to the word vector (see Figure 2).

**Figure 3:**
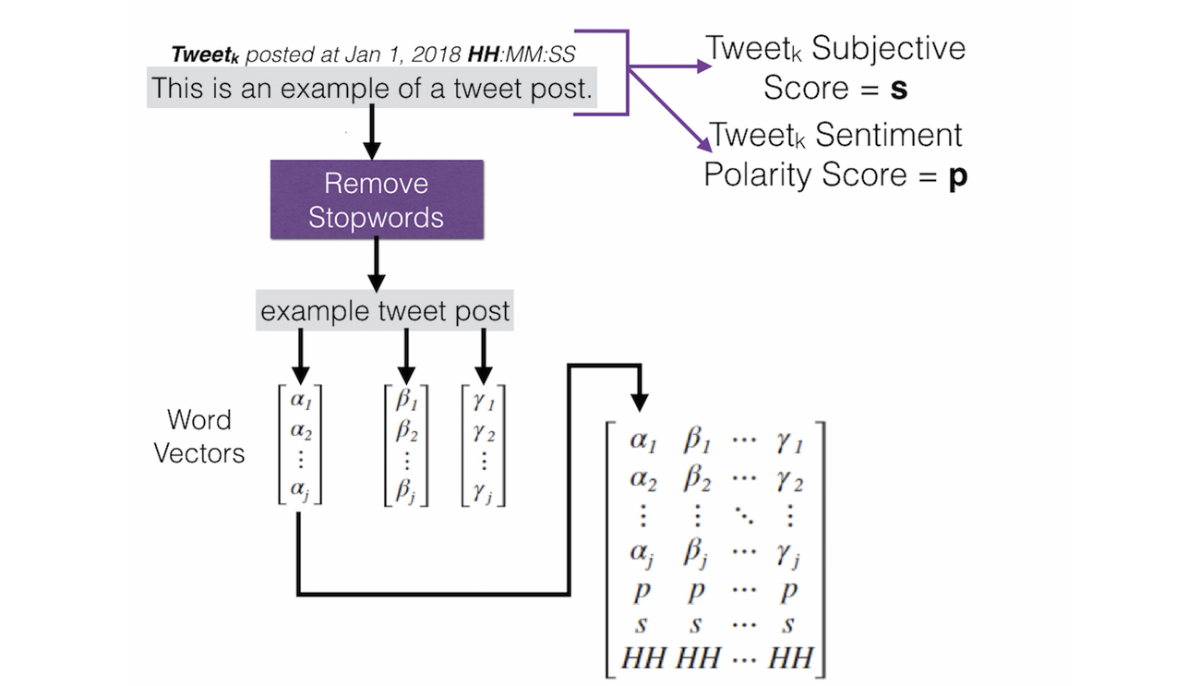
Example tweet converted to the matrix format that represents two dimensions of the input tensor. This example will represent what will be the slice of our input tensor labeled as tweet_k_ in Figure 2. The stopwords (ie, words that have no meaning such as the and a) are removed thus only three words remain. Each word has a word vector associated with it from a trained continuous bag-of-words model. Lastly, word vector representation is concatenated with a holistic tweet feature: the sentiment polarity, subjectivity, and hour of post.

To elaborate further, every row of the input matrix I represents a tweet without stop words (ie, words that give no meaning such as *the* and *a*) and non-English characters and each word in that tweet was represented by a continuous bag-of-words (CBOW) word vector [35] that extends along the third dimension (see Figure 3). The next layer in the third dimension was the sentiment polarity where the range (0,+1) is a positive tweet, (−1,0) is a negative tweet, and 0 is a neutral tweet. In addition, the next layer is the tweet’s subjectivity that ranges from 0 to 1 where 0 is very objective and 1 is very subjective. Both the sentiment and the subjectivity were calculated using the TextBlob sentiment analysis library [36]. The last layer, the third dimension, is the hour of the tweet post in a 24-hour representation (HH). Finally, if a tweet has fewer words within it than κ, then the rest of the row is filled with zeros.

## Results

### Experiment Setup and Data

All tweets in this study were gathered from the UCLA first-year student study and were labeled using the simple linear model created from their Basis band data (described in the Data Preprocessing subsection). Each word in the UCLA first-year students’ tweets was associated with a word vector; however, the freshmen Twitter dataset was not large enough to create a semantically accurate word vector representation. Therefore, all word vectors were trained on a larger Twitter dataset from the University of California, Irvine [37], covering a duration of 7 months and consisting of 720 million tweets, 6.6 billion words, and 3.9 million unique words. The word vectors were trained using the CBOW Word2Vec algorithm, with a dimension of 100 and a window of length 5. In addition, rare words, occurring fewer than 5 times, were removed.

The classification algorithm had 18,394 labeled UCLA freshmen’s tweets, where 8,068 were sleep deprived tweets and 10,326 were sleep-sufficient tweets. The maximum sentence size with Spanish and English stop words removed was 34. The training/validation and testing dataset were split 90/10 and the training and validation dataset were split from the previous 90 into another 90/10 split. Finally, the GRU’s dense layer had a dropout rate of 0.5 for generalization purposes.

To keep from misclassifying tweets, it was assumed that a participant must sleep within 28 hours of their last new-day sleep. This threshold of 28 hours was determined based on the 2017 Accreditation Council for Graduate Medical Education regulation of a maximum shift length. This threshold seemed reasonable because studies are performed to create this threshold for medical students, and it was assumed that a freshman student would not stay awake (even if it is only for a 15-min nap) any longer than this maximum shift length. Therefore, if the sensor exhibited a student staying awake for over 28 hours, this student is showing signs of missing data, and every tweet after that 28th hour and before the next new sleep is removed from the dataset.

### Experiment Results

The final metric of success was calculated using the area under the receiver operating characteristic curve (AUC) to get a fair comparison of the imbalanced dataset where 0.5 is a performance metric as good as random. With the above parameters, the GRU showed the best results, with an average AUC of 0.68 with 0.003 SD. This result shows promise that it is possible to identify when a student is sleep deprived based solely on their Twitter posts.

## Discussion

### Principal Findings

The findings suggest the feasibility of integrating wearable device data and social media data to monitor sleep deprivation.

We identified 2 key points from this study. First, for researchers to be able to use wearable devices as a method of monitoring tiredness, models need to be created that can use wearable devices to monitor sleep quality and quantity to identify when a person is at a high TL to be considered sleep deprived. As, to our knowledge, there are no current numerical models tracking a person’s real-time levels of tiredness, there is a lack of ground truth determining when a person is sleep deprived. This study defines tiredness using a simple linear model based on the student’s Intel Basis band data to create data labels for the student’s tweets. Future researchers can build on this attempt to model tiredness to more accurately identify TLs.

This study is one of the first to integrate multiple sources of remote data, including social media, self-reported Web-based surveys, and Intel Basis band data. We integrate these different data sources to develop a tiredness model and to passively monitor sleep deprivation from nonsensor devices such as social media. Results suggest the feasibility of using wearable smart watches and social media (ie, Twitter) data for monitoring sleep deprivation among undergraduate students. We found, in our study, that we were able to train and test a model that used Twitter data and could predict student’s objective level of sleep, as measured by a sleep monitoring device. Therefore, because of the popularity of social media and the predicted accuracy of the results, a novel technique to assess global sleep sufficiency and deprivation has been shown.

### Limitations and Future Work

Although our early results look promising, there are limitations to this study. First, because of the requirements that the participating student must have worn their sleep-tracking device, taken a Web-based survey, and tweeted at least 3 times per week, this pilot feasibility study was limited to a final sample size of 86 out of 197 freshmen students. Second, data were only recorded during the fall semester, thus limiting this classification model to a specific demographic in a specific time window. In the future, research can expand upon this work to include an entire school year and across several other universities to obtain more generalizable sleep-deprivation results.

In addition, we recognize that student populations have different behaviors and related sleep factors (eg, feeling the need to stay up all night to study for tests) that can affect tiredness and sleep deprivation differently from other occupations, thus reducing the ability to generalize these findings to all people and populations.

A further limitation to this study is that we used Twitter as the only social media platform. Participants were screened to include those who frequently use Twitter because Twitter is a frequently used data source in modeling research. It is possible that people tweet differently than they use Instagram or Facebook, so there may be signs of sleep deprivation that were missed because other social media platforms were not used in this study. This question can be studied in future research and has been added as a recommendation for future research.

It is also noted that it is possible that students changed their Twitter behavior because they had knowledge of being observed. Therefore, another limitation in this study is that we have no way to know whether a student varied their language based on being observed or not; as a result, it is assumed that the language was not altered. The study of how sleep-deprived and sleep-sufficient language changes when being observed or not observed is left for future research.

In addition, more research needs to be performed in verifying the device and identifying its average error as we used bands provided to us and did not conduct validation methods to assess the accuracy of the readings for each band. Furthermore, there was no metric available to determine how tired a person is throughout every minute of their day; therefore, a simplistic model was created to best define a student’s tiredness based upon their previous night’s sleep. This model limits the accuracy of the classification model through the numeric model’s rough approximation of the sleep-deprived period. Similarly, sleep deprivation is subjective (ie, people have differing levels of sleep needed), making it difficult to evaluate sleep deprivation objectively. We attempted to address this issue by validating the study participant data by corresponding one night or less a week wearable (objective) data to a weekly survey (subjective) measurement asking how the student slept the night before. Future research can build and improve on these methods for more accurate classification.

Finally, the numeric model was limited to only including sleep quantity to determine the beginning tiredness/sleep-deprivation level of the student in the morning. This model could be further improved by incorporating sleep quality, types of beverages, and food consumed throughout the day; types of medications taken; and exercise duration and type into the model. For determining tiredness at the beginning of the student’s day, sleep quality could be quantitatively measured using data captured by smart wearables. Studies have shown that the number of interruptions in sleep and the percentage of each sleep stage throughout the night determine the sleep quality [38]. However, more research needs to be performed to numerically model sleep quality and determine how it affects the TL variance throughout the day.

### Conclusions

This pilot study suggests the feasibility of (1) modeling a student’s TL throughout the duration of their day from smart wristband devices and (2) determining whether a student is sleep deprived based on their social media behavior. Future research should further explore the integration of multiple data sources to monitor real-time changes in tiredness.

## Abbreviations

AUC: area under the receiver operating characteristic curve
CBOW: continuous bag of words
GRU: gated recurrent unit
SLD: sleep deprived
TL: tiredness level
UCLA: University of California Los Angeles
